# A Retrospective Review of Endogenous Endophthalmitis: Three Years of Experience at Sultan Ahmad Shah Medical Centre at International Islamic University Malaysia

**DOI:** 10.7759/cureus.63175

**Published:** 2024-06-26

**Authors:** Nur-Syifa Athirah Qistina Alias, Mohd-Fadzil Abu-Bakar, Abdul-Hadi Rosli, Aidila Jesmin Jabbari

**Affiliations:** 1 Ophthalmology, Kulliyyah of Medicine, International Islamic University Malaysia, Kuantan, MYS

**Keywords:** pars plana vitrectomy (ppv), intravitreal injections, bilateral endophthalmitis, bacterial endogenous endophthalmitis, klebsiella endophthalmitis, fungal endogenous endophthalmitis, acute endophthalmitis

## Abstract

Introduction

Endogenous endophthalmitis is characterized by severe intraocular inflammation caused by the invasion of microorganisms into the anterior and posterior chambers of the eye. It results from hematogenous spread from distant foci of infection. This, in turn, leads to potential vision loss and blindness due to reduced anatomical and functional outcomes. The latest reported prevalence of endogenous endophthalmitis accounts for at least 2-8% of cases of general endophthalmitis which is fairly significant.

Purpose

This study aimed to analyze the clinical profile of endogenous endophthalmitis presented in the Ophthalmology Clinic, Sultan Ahmad Shah Medical Centre at International Islamic University Malaysia (SASMEC@IIUM). This study includes the patients' demographics, clinical manifestations, causative organism, treatment, and final visual outcome.

Methods

This is a retrospective case series of endogenous endophthalmitis patients from January 2020 to June 2023. The data were obtained from the patients' medical records in SASMEC@IIUM.

Results

A total of six patients (six eyes) were diagnosed with endogenous endophthalmitis from January 2020 to June 2023. Four patients (66.6%) were female, with a mean age of 51.6 ± 17.5 years. Presenting visual acuity ranged between 6/21 to hand movement (HM). Five patients (83.3%) presented with reduced vision, while one presented with eye redness (16.6%). Ocular signs included vitritis and retinitis (five eyes, 83.3%), hypopyon (five eyes, 83.3%), injected conjunctiva (five eyes, 83.3%), and eyelid swelling (one eye, 16.6%). The most common primary infection seen was intraabdominal sepsis (three patients, 50%), septic arthritis, hospital-acquired pneumonia (HAP), and urinary tract infection (UTI). Vitreous biopsy was only positive in two patients (33.3%) However, five out of the six patients (83.3%) had positive blood cultures (two *Staphylococcus aureus*, two *Klebsiella pneumoniae* and one *Pseudomonas aeruginosa*). All patients received intravitreal injections and intravenous antibiotics. Only one patient underwent subsequent trans pars plana vitrectomy (TPPV). Final visual acuity ranged from 6/6 to no light perception (NPL).

Conclusion

In this case series of six patients, we observed a variety of outcomes with similar presentations despite standardized treatment in all patients. Five out of six patients showed poorer visual outcomes and only one patient showed a final visual acuity of 6/6. Therefore, further study with a larger sample size is needed to evaluate the factors associated with the final visual outcome in endogenous endophthalmitis.

## Introduction

Endophthalmitis is a catastrophic ocular condition characterized by severe intraocular inflammation caused by the invasion and seeding of microorganisms into the anterior and posterior chambers of the eye. This fulminant ocular infection, in turn, leads to potential visual loss and blindness due to reduced anatomical and functional outcomes [1,2].

Endophthalmitis usually manifests as acute or chronic conditions, and the mode of spread is further subdivided into exogenous or endogenous types. Exogenous endophthalmitis occurs following the direct introduction of pathogens into the eye (due to trauma or following surgical procedures), while endogenous endophthalmitis results from hematogenous spread from distant foci and is associated with secondary systemic pathology [3]. The main risk factors for developing endogenous endophthalmitis are medical factors such as diabetes mellitus and immunocompromised individuals [4].

In general, endogenous endophthalmitis is relatively uncommon, with a reported global prevalence of 2-8%. It leads to bilateral eye infection in 15-20% of cases, and 50% of endogenous endophthalmitis cases are frequently associated with fungal infections according to a study by Novosad et al. [5].

Prompt diagnosis and immediate management of endogenous endophthalmitis is crucial to minimize the extension of ocular damage and preserve visual function [6]. However, the course of endophthalmitis, treatment success, and visual outcome are still uncertain due to the virulence factor of the organism, the immunocompromised state of the host and diagnostic dilemma [5]. To date, diagnosing endogenous endophthalmitis is still a challenge for physicians as the clinical presentations vary. It is reported that diagnosis error or delayed diagnosis during first presentation occurs in at least 25-33% of the cases, leading to delayed treatment initiation [7].

A favorable visual outcome has been demonstrated by early intravitreal and systemic antibiotics only without requiring vitrectomy [4]. Despite the prompt initiation of topical and systemic antibiotics, vitrectomy still plays a vital role in managing endogenous endophthalmitis [2].

Considering the variability and uncertainty in the nature of the disease, more information regarding the clinical profile of endogenous endophthalmitis would be an extremely valuable added value in understanding the management of endogenous endophthalmitis.

This study aimed primarily to describe and report the common clinical profile of endogenous endophthalmitis in Sultan Ahmad Shah Medical Centre at International Islamic University Malaysia (SASMEC@IIUM) by describing the demographic data and pathogenesis, review of clinical features, description of pathogenesis, summarizing the treatment, outline the complications, and visual outcome of endogenous endophthalmitis after treatment. The secondary aim was to identify the best clinical approach to managing endogenous endophthalmitis.

## Materials and methods

Study design

This retrospective case series was conducted in SASMEC@IIUM, a newly established tertiary teaching hospital in Kuantan, Pahang, Malaysia. The data were obtained from the patients' electronic medical records (EMR) in SASMEC@IIUM from January 2020 to June 2023. This study was approved by the Ethics Committee of Kulliyyah of Medicine, IIUM (ID number IREC 2023-165) and was carried out in accordance with the Declaration of Helsinki. Informed consent was renounced due to the retrospective nature of this study.

Participants

Patients diagnosed with endogenous endophthalmitis in SASMEC@IIUM were identified by reviewing the patient database available in the EMR. Patients diagnosed with endogenous endophthalmitis were selected based on history, clinical presentation, and positive blood culture results. Inclusion criteria included all patients who were referred to the Ophthalmology Department as inpatient referrals and were diagnosed with endogenous endophthalmitis within the time frame. Patients were excluded if the patient was diagnosed as having exogenous endophthalmitis, uveitis, or other causes of ocular infections.

Data collection

Data from the patient's EMR with keyword of "endogenous endophthalmitis" based on the International Classification of Disease, 10th revision (ICD-10) was reviewed and collected. General demographic features (age, gender, race), underlying comorbidities and risk factors, clinical presentation, baseline visual acuity, intraocular pressure (IOP), slit lamp examinations, microbiologic report of the causative organism through blood, aqueous or vitreous sample, treatment modalities, and final visual outcome were documented. Visual outcomes were evaluated based on best-corrected visual acuity (BCVA) and at the latest follow-up ranges from one month to 12 months period after completion of treatment. Two groups were identified: a group with improved visual outcome from baseline and another group with no improvement or deterioration from baseline visual acuity.

Microbial examinations were conducted under the Pathology Department at SASMEC@IIUM. Vitreous samples were obtained through aspirated vitreous tapping or during trans pars plana vitrectomy (TPPV). Positive microbial diagnosis was confirmed through vitreous samples sent for gram-staining, culture, and sensitivity. 

## Results

Case presentations

Case 1

**Figure 1:**
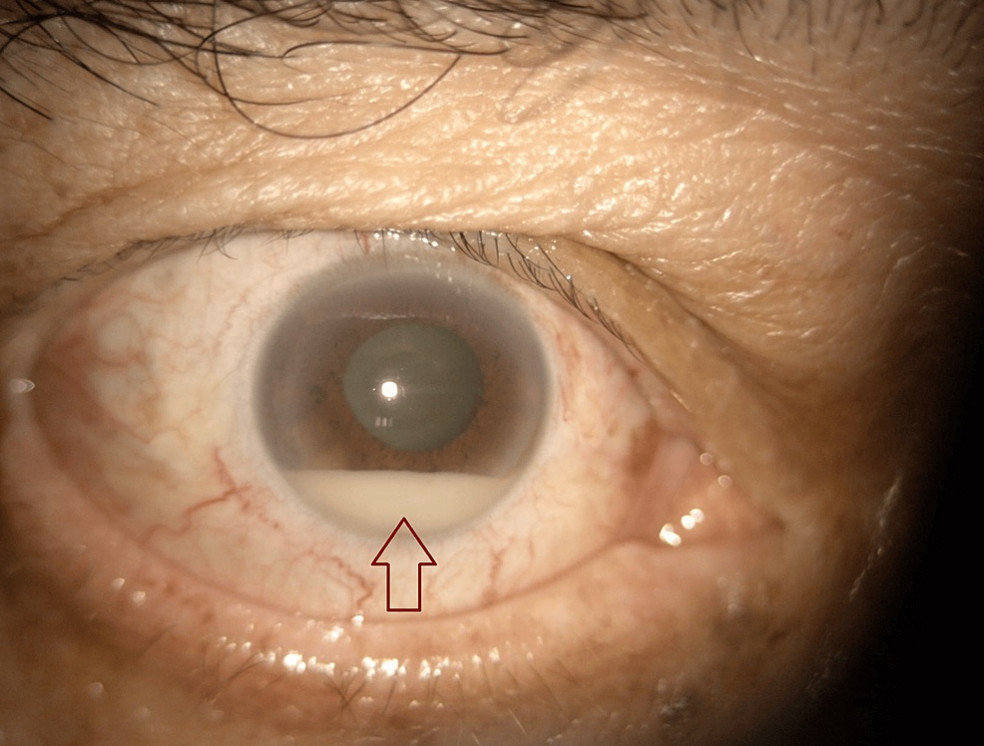
Right anterior segment photo showing hypopyon (red arrow).

**Figure 2:**
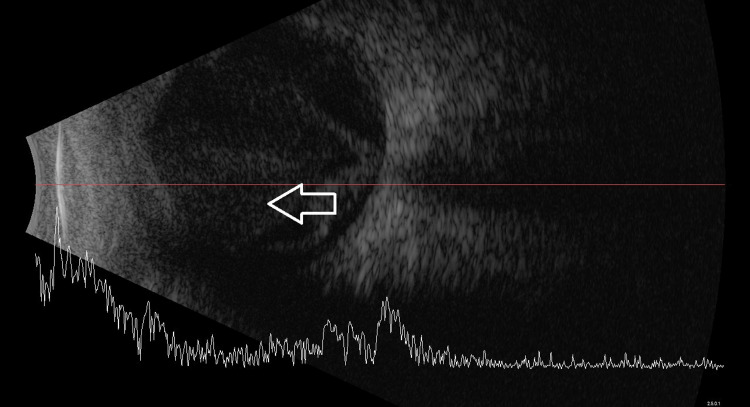
B-scan showing dense vitritis (white arrow).

A 72-year-old gentleman with underlying poorly controlled diabetes mellitus (DM), hypertension (HPT), and atrial fibrillation complained of sudden onset, painful right eye blurring of vision for two days after one week of admission under the medical team for right shoulder septic arthritis. The visual acuity of the right eye was light perception (LP) and the left eye was 6/15. The ocular examination revealed right eyelid swelling with injected conjunctiva. There was presence of anterior chamber cells and hypopyon (Figure 1). The fundus examination showed hazy vitreous with a limited view of the retina. Ultrasound B-scan showed dense vitritis (Figure 2). A diagnosis of right eye endogenous endophthalmitis was made. Blood culture and vitreous sample through vitreous tapping were sent for gram stain as well as culture and sensitivity. Immediate intravitreal vancomycin (1mg in 0.1ml) and intravitreal ceftazidime (2mg in 0.1ml) were given in the same setting. The patient also was started on topical moxifloxacin 0.5% and systemic IV ciprofloxacin 400mg three times daily. Blood culture was positive for *Staphylococcus aureus*, however vitreous culture showed no organism. The patient was counseled for TPPV, however he refused. Therefore, he underwent multiple intravitreal antibiotic injections until the infection was controlled. His final right visual acuity deteriorated to no light perception (NLP) as the patient developed phthisical eye.

Case 2

**Figure 3:**
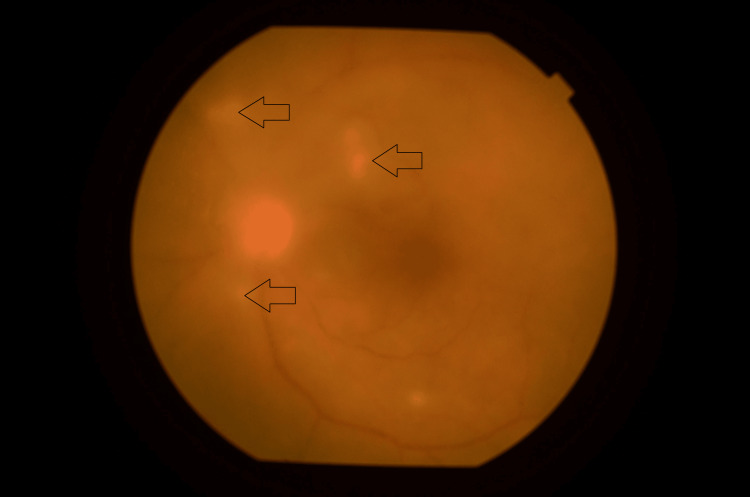
Left fundus photo showing vitritis and multiple retinitis lesion (black arrow).

A 56-year-old lady with underlying poorly controlled DM and HPT complained of left eye worsening vision for the past eight days. The patient noted deterioration of her vision in the ward, while she was treated for liver abscess. Liver tissue culture yielded positive for *Klebsiella pneumoniae *(sensitive to gentamicin and trimetho/sulfamethox). The visual acuity of the right eye was 6/6 and the left eye was 6/21. Anterior segment examination revealed anterior chamber cells with hypopyon. Fundus examination showed vitritis with multiple retinitis lesions (Figure 3). The IOP over the left eye was 40 mmHg. A diagnosis of left-eye endogenous endophthalmitis was made. Immediate intravitreal tap was performed, and a vitreous sample was sent for culture and sensitivity. Intravitreal vancomycin (1mg in 0.1ml) and intravitreal ceftazidime (2mg in 0.1ml) were given in the same setting. Blood culture and vitreous culture were negative. The patient was started on topical moxifloxacin 0.5% and systemic IV ciprofloxacin 400mg three times daily. The patient was also given topical IOP lowering agents which were topical Simbrinza (combination of brinzolamide and brimonidine tartrate) twice daily, topical travaprost ON and topical timolol twice daily. Subsequently, the patient underwent TPPV after being given one intravitreal injection. Post TPPV, the left eye visual acuity was improved to 6/7.5. Unfortunately, she developed recurrent endogenous endophthalmitis six weeks post-operative requiring repeated TPPV. The final visual acuity of the left eye was 6/30.

Case 3

**Figure 4:**
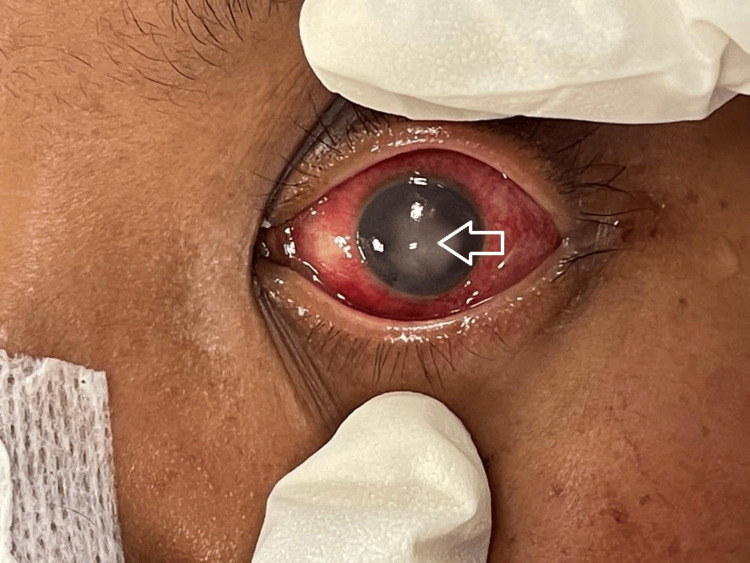
Left anterior segment showing chemosed and injected conjunctiva with corneal opacity and hypopyon (white arrow).

A 31-year-old lady was undergoing treatment for intrabdominal sepsis post laparoscopic salpingectomy with hospital-acquired pneumonia (HAP) requiring ventilation support in the intensive care unit. She was initially referred for left eye redness associated with copious discharge. Anterior segment examination of the left eye revealed injected conjunctiva with corneal opacity and hypopyon (Figure 4). Blood culture revealed multidrug-resistant Pseudomonas aeruginosa (resistance to ceftazidime, ciprofloxacin, cefepime, gentamicin, imipenem and meropenem). During referral patient was already on intravenous infusion vancomycin 1000mg. A diagnosis of left-eye endogenous endophthalmitis was made. Immediate intravitreal tap was performed, and the vitreous sample was sent for culture and sensitivity. Intravitreal vancomycin (1mg in 0.1ml), ceftazidime (2mg in 0.1ml) and amphotericin B (5 mcg in 0.1ml) were given. The patient also started on topical moxifloxacin 0.5%, topical ceftazidime 5% and topical amphotericin B 0.25%. No growth was cultured from the vitreous sample. TPPV was not performed as the patient was hemodynamically unstable. The patient unfortunately passed away during the course of treatment.

Case 4

**Figure 5:**
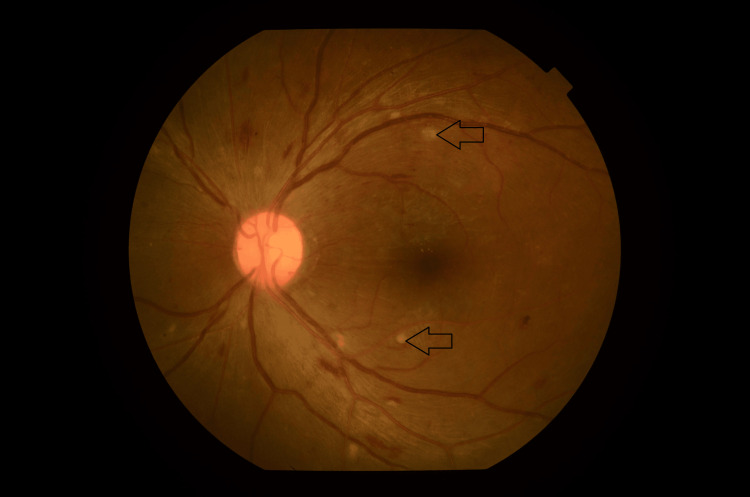
Left fundus photo showing multiple retinitis lesions over superotemporal arcuate and inferotemporal arcuate (black arrow).

A 31-year-old lady with underlying DM and HPT presented with progressive painless blurring of vision for the past two days during her stay in the intensive care unit. She was admitted for Guillain-Barre syndrome with HAP. The visual acuity of the right eye was 6/36 and the left eye was 6/7.5. The right anterior segment examination revealed injected conjunctiva with anterior chamber cells and hypopyon. The right fundus showed presence of vitritis peripherally and multiple retinitis lesions over superotemporal arcuate and inferotemporal arcuate (Figure 5). A diagnosis of right eye endogenous endophthalmitis was made. Immediate intravitreal tapping commenced, and a vitreous sample was sent for culture and sensitivity. Intravitreal vancomycin (1mg in 0.1ml) and ceftazidime (2mg in 0.1ml) were given during the same procedure. Blood culture was positive for S*taphylococcus aureus* while the vitreous sample resulted in no growth. The patient also started on topical moxifloxacin 0.5% and systemic IV cloxacillin 2g four hourly. The patient's ocular condition improved remarkably after the first intravitreal antibiotic injection. The final visual acuity was 6/6 without episode of recurrence infection.

Case 5

**Figure 6:**
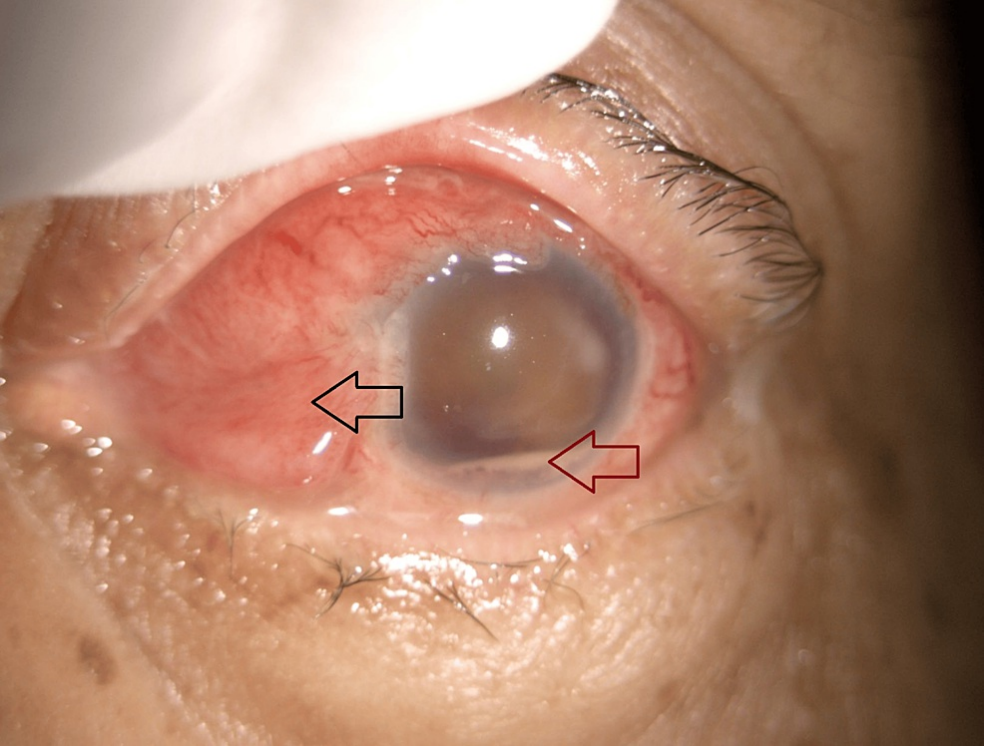
Left anterior segment showing chemosis and injected conjunctiva (red arrow) and hypopyon (black arrow).

A 63-year-old lady with underlying poorly controlled DM presented with left eye redness and reduced vision for seven days. It was associated with eye discharge and eyelid swelling. One week prior to this presentation, the patient had a history of urinary tract infection (UTI) which was treated with oral antibiotics prescribed by the general practitioner. The relative afferent pupillary defect (RAPD) was positive over the left eye. The presenting visual acuity of the right eye was 6/12 and the left eye was LP. The left anterior segment showed injected conjunctiva with hazy cornea. There was presence of severe anterior chamber inflammation with hypopyon as well (Figure 6). Fundus examination revealed severe vitritis with no view of the retina. The IOP of the left eye was 54 mmHg. Ultrasound B-scan revealed dense vitritis with diffuse choroid and scleral thickening. The diagnosis of left endogenous panophthalmitis was made. In view of the presenting visual acuity of the left eye as NPL, the patient was counselled that the aim of treatment was to salvage the globe. Immediate intravitreal tapping commenced, and a vitreous sample was sent for culture and sensitivity. Intravitreal vancomycin (1mg in 0.1ml) and ceftazidime (2mg in 0.1ml) were given during the same procedure. The patient started on topical moxifloxacin 0.5% and systemic IV ciprofloxacin 400mg three times daily. The patient was also given topical IOP lowering agents which were topical Simbrinza (combination of brinzolamide and brimonidine tartrate) twice daily and topical Duotrav (combination of timolol and travoprost) ON. The vitreous sample for culture and sensitivity was positive for *Escherichia coli*. However, the blood and urine samples were negative. The patient received a total of three intravitreal antibiotics injections (amikacin was added in subsequent injection) every 48 - 72 hours. The final visual acuity remained NLP.

Case 6

**Figure 7:**
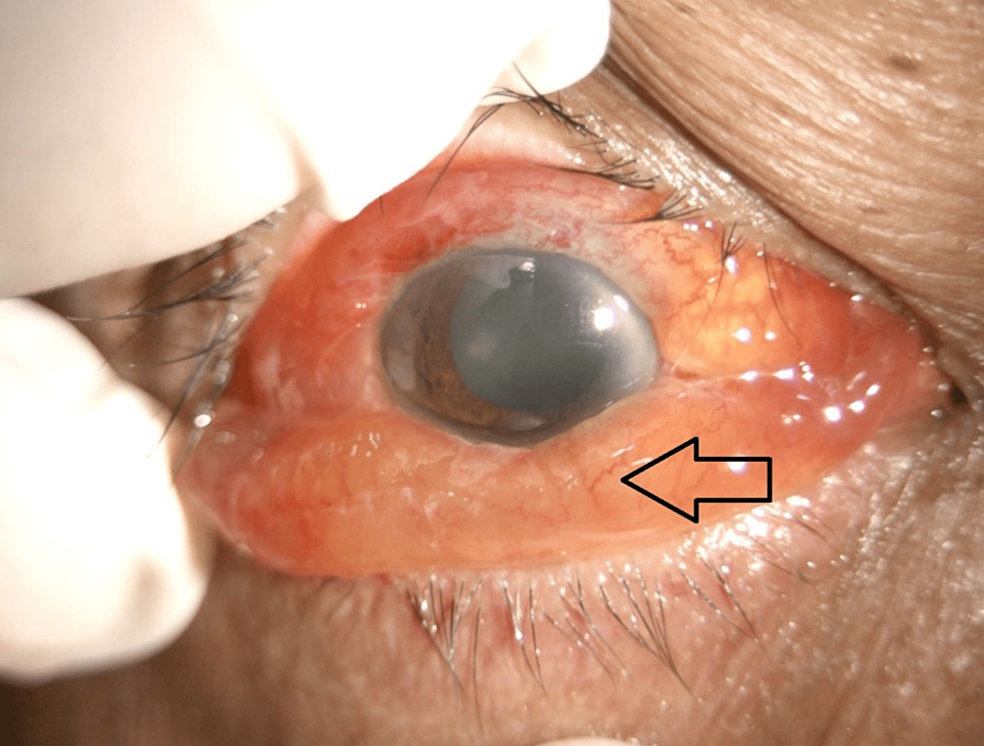
Right anterior segment taken during ongoing treatment showing injected conjunctiva, severe chemosis with resolved hypopyon (black arrow).

A 63-year-old gentleman with underlying diverticulitis with sigmoid stricture complained of right eye painful blurring of vision for two days during his admission in ward. The patient was admitted to the medical ward for intraabdominal sepsis. The visual acuity of the right eye was hand movement (HM) and left eye was 6/6. The right anterior segment revealed injected conjunctiva with presence of anterior chamber inflammation (Figure 7) and hypopyon. Fundus examination showed vitritis and multiple retinitis lesions. A diagnosis of right eye endogenous endophthalmitis was made. Immediate intravitreal tapping commenced, and a vitreous sample was sent for culture and sensitivity. Intravitreal vancomycin (1mg in 0.1ml), ceftazidime (2mg in 0.1ml) were given as well. Topical moxifloxacin 0.5% and systemic IV ceftazidime 2g three times daily was started. The patient was counseled for TPPV but he refused. Repeated intravitreal antibiotic injection was given after 48 hours. The final visual acuity of the patient did not improve and was maintained as HM. The patient subsequently opted for at-own-risk discharge after one week of receiving IV ceftazidime.

Demographic data

A clinical summary of six patients (six eyes) (cases 1-6) who presented to SASMEC@IIUM were identified with endogenous endophthalmitis within the study period between January 2020 and June 2023. The mean age of the patients was 51.6 ± 17.5 years (cases 1-6), with slight female predominance seen (four patients, 66.7%) (cases 2-5). Initial presenting visual acuity ranged between 6/21 to HM (cases 1-6). One patient had no documentation of visual acuity as the patient was ventilated in the ICU at the time of referral (case 3). One patient was initially referred to in the ICU setting while others were referred during their inward stay (case 3). All examinations were done under slit lamp examinations, while only one patient was examined in ward as patient was intubated in the ICU (case 3). All patients were initially offered TPPV, however only one patient proceeded with TPPV (case 2).

Systemic comorbidity 

Three patients (50%) had known comorbidities (cases 1,2,4), including poorly controlled diabetes mellitus and hypertension, with predisposing factors such as liver abscess and shoulder septic arthritis (case 1 and case 2). One patient (16%) had a history of diverticulitis (case 6), and another two patients (33.3%) had no known medical illness (case 3 and case 5) before diagnosis of endogenous endophthalmitis (Table 1).

**Table 1:** Summary of clinical characteristics of six clinical cases. BCVA: best-corrected visual acuity, DM: diabetes mellitus, HPT: hypertension, LP: light perception, HM: hand movement, NLP: no light perception, IVT: intravitreal antibiotics (combination of intravitreal vancomycin, ceftazidime; case 3 also received intravitreal amphotericin B), TPPV: trans pars plana vitrectomy, blood: blood culture and sensitivity, tissue: tissue culture and sensitivity, ocular: vitreous culture and sensitivity.

Case	Sex	Risk Factor	Isolate (blood/ocular/tissue)	Organism	Treatment	Initial BCVA	Final BCVA
1	Male	DM, HPT, Septic shoulder arthritis	Bacterial (blood)	Staphylococcus aureus	IVT	LP	NLP
2	Female	DM, HPT, Liver abscess	Bacterial (tissue)	Klebsiella pneumoniae	IVT & TPPV	6/12	6/30
3	Female	Intraabdominal sepsis	Bacterial (blood)	Pseudomonas aeruginosa	IVT	Intubated	Death
4	Female	DM, HPT	Bacterial (blood)	Staphylococcus aureus	IVT	6/38	6/6
5	Female	Urinary tract infection	Bacterial (ocular)	Escherichia coli	IVT	LP	NLP
6	Male	Diverticulitis, Occult sepsis	Bacterial (blood)	Klebsiella pneumoniae	IVT	HM	6/120

Primary source of infection

The most common primary infection seen was intraabdominal sepsis (three patients, 50%) (cases 2,3,6) followed by septic arthritis (case 1), HAP (case 4), and UTI (case 5) (one patient each, 16.7%) (Table 2).

**Table 2:** Primary source of endogenous endophthalmitis. The data has been represented as N (number of patient) and % (percentage).

Primary source	Total (N=6)
Shoulder septic arthritis	1 (16.6%)
Liver abscess	1 (16.6%)
Intraabdominal sepsis	2 (33.3%)
Hospital acquired pneumonia	1 (16.6%)
Urinary tract infection (UTI)	1 (16.6%)

Ocular presentations

All the cases in our series had only unilateral eye involvement predominantly the left eye (four eyes, 66.7%) (cases 2,3,5,6). The timing of the first clinical complaint or sign to the time of referral ranged from two days to seven days (cases 1-6). Five patients (83.3%) (cases 1,2,4,5,6) presented with reduced vision as their main complaint. One patient ventilated in the ICU was referred for eye redness (16.6%) (case 3). Ocular signs include vitritis and retinitis (five eyes, 83.3%) (cases 1,2,4,5,6), hypopyon (five eyes, 83.3%) (cases 1-5) and injected conjunctiva (five eyes, 83.3%) (cases 2-6). Fellow eyes were unremarkable in all the patients (six patients, 100%) (cases 1-6).

Microbiology findings

Vitreous fluid obtained in one patient (16.6%) showed positive microbial vitreous cultures, *Escherichia coli* (case 5). At the same time, five patients (83.3%) reported positive blood cultures (cases 1,2,3,4,6). Only one patient (16.6%) interestingly had a negative blood culture with positive vitreous culture and sensitivity (case 5). The organism included two positive cultures for *Klebsiella pneumoniae* (33.3%) (case 2 and case 6), two positive cultures of *Staphylococcus aureus* (33.3%) (case 1 and case 4), and one for multidrug-resistant* Pseudomonas aeruginosa* (16.6%) (Table 3) (case 3).

**Table 3:** Microbial isolate from blood and vitreous sample. The data has been represented as N (number of patient) and % (percentage).

Species	Total (N)	Percentage (%)
Culture positive		
Blood	5	83%
Vitreous	2	33%
Culture negative		
Blood	1	16%
Vitreous	4	66%

Management and outcomes

All patients (cases 1-6) received between one to three intravitreal injections (a combination of intravitreal vancomycin 1mg/0.1ml and intravitreal ceftazidime 2.2mg/0.1ml and systemic antibiotics such as intravenous ciprofloxacin 400 mg three times daily. However, only one patient agreed to TPPV (case 2). After three months of follow-up, final visual acuity ranged from 6/6 to NPL (cases 1-6). As for the final ocular outcome, two patients observed improvement in the final visual acuity (case 4 and case 6), another three patients had deteriorated in the final visual acuity compared to baseline (cases 1,2,5), and one patient passed away during the course of treatment due to sepsis (case 3).

## Discussion

Endogenous endophthalmitis is a rare complication whereby there is a hematogenous spread of infection to the choroid, that destroys the retina and devastates the vision [6,8]. It has a rapid clinical course requiring prompt diagnosis and management to preserve the patient's visual potential [4]. However, to date, it still imposes a diagnostic challenge for the clinician [8]. This review aims to analyse and compare the clinical profiles and outcomes of endogenous endophthalmitis at our tertiary hospital in Malaysia from those reported in other countries.

In our study, the clinical profile of endogenous endophthalmitis did not show any specific preferences towards age, with a mean age of 51.6 ± 17.5 years (range from 31 to 72 years old). It is shown that it occurs in both genders, with a slight female preference (66.7%) compared to males. Interestingly, other studies conducted by Yoshida et al. and Gajdzis et al. supported no age and sex predilection towards the incidence of endogenous endophthalmitis [6,9], in contrast to our research, which shows more female predilection. However the relationship contributing to the incidence of endogenous endophthalmitis remains controversial and unclear. Uniocular involvement with predominant left eyes was seen in four cases (66.7%) in this study. According to a worldwide review done on endogenous endophthalmitis, uniocular involvement was seen more frequently despite being caused by blood-stream-related infections especially in bacterial infections compared to fungal infections [10,11], and many recent reviews confirmed the same outcome as our study: the left eye was more common than the right eye [11-13].

Endogenous endophthalmitis is usually associated with predisposing factors, mainly patients with chronic metabolic diseases such as diabetes mellitus, immunocompromised patients, and intravenous drug users [6,10,14,15]. Our study showed good concurrence with three out of the six patients (50%) having poorly controlled or newly diagnosed diabetes mellitus and five of the six having pre-existing infection. Generally, patients with more comorbidities are shown to have a poorer visual recovery prognosis.

The most common primary source of infection identified was intraabdominal sepsis (two patients, 33.3%). Other causes included liver abscess, septic arthritis, HAP, and UTI. In contrast to this review, two other studies conducted at a tertiary center in Malaysia reported UTIs as the most common primary source of infection [12,16]. The type of microorganisms and primary infection may differ accordingly despite reporting in similar settings. Limitations of reported cases in different center may also cause slight differences in the primary source reporting.

Five patients (83.3%) presented with reduced vision. One patient was referred for eye redness (16.6%). Ocular signs include vitritis and retinitis (five eyes, 83.3%), hypopyon (five eyes, 83.3%), injected conjunctiva (five eyes, 83.3%), and eyelid swelling (one eye, 16.6%). Two studies conducted in Korean tertiary hospital and Malaysia tertiary hospital documented similar initial presentations to our study, including mainly blurring of vision, eye redness, and eye pain as the primary ocular complaint prior to diagnosis of endogenous endophthalmitis duration ranging from one week to one month from the time of complaint to establishment of diagnosis [16,17]. Most of our patients presented with symptoms of less than two weeks, however their final visual outcome varied. This could be due to other confounding factors such as severity of the infection, virulency of the microorganism and associated comorbidities of the patients, especially those who have poorly controlled diabetes mellitus.

The definitive diagnosis of endogenous endophthalmitis is established with positive microbiologic evidence from intraocular samples, namely the aqueous or vitreous sample. Extraocular samples such as blood cultures or other sites can also highly support the diagnosis of endogenous endophthalmitis [16]. In our review, a total of five patients (83%) showed positive microbial obtained through blood culture and sensitivity with only two patients (33%) having culture-positive vitreous. This proved that blood culture and sensitivity yielded better values compared to vitreous samples. Previous literature reviews also supported the importance of obtaining a blood culture before the commencement of treatment to establish the clinical diagnosis, as shown in our study [11,18]. In contrast, two studies conducted by Ness et al. and Binder et al. projected a high positivity rate of 81% and 70.5% with intraocular samples (aqueous or vitreous) compared to blood samples [19,20].

Previous published reviews conducted in Australia, Germany, and Miami Hospital reported a higher incidence rate of fungal infection than bacterial infection [8,20,21]. However, all of our patients (100%) had bacterial infection, predominantly by gram-negative bacteria (four eyes, 66.7%). None of our patients were diagnosed with fungal endogenous endophthalmitis. We presumed it to be due to the limitations in our study caused by the low number of reported endogenous endophthalmitis cases. Multiple other studies seconded that causative organism varies according to country, whereby Asian countries are associated more with bacterial infection than Western countries, revealing more fungal-related infections [16]. We reported two cases of gram-positive pathogens, namely gram-positive *Staphylococcus aureus*, while gram-negative bacteria in our study showed two positive cases of *Klebsiella pneumoniae* and one case of *Escherichia coli* and *Pseudomonas* species. The result of our review is consistent with an East Asian study by Wong et al., which shows that gram-negative organisms are more common, with *Klebsiella* species responsible for 60% of bacterial endogenous endophthalmitis cases [22]. Another study conducted in the USA found that *Klebsiella pneumoniae* may contribute to 15 times more prevalence of endogenous endophthalmitis, which is similarly reported in our study [23]. Recently, it has been reported that liver abscess is significantly associated with *Klebsiella pneumoniae*, especially in Asian countries. Thus, extra care and monitoring should be given to avoid the increasing prevalence of endogenous endophthalmitis [24]. Zenith et al. reported that 50% of patients diagnosed with *Klebsiella* endogenous endophthalmitis resulted in enucleation as the endpoint due to a high virulence rate and poor prognosis [18]. *Streptococcus pneumoniae* is a rare cause of endogenous endophthalmitis and is associated with a poor prognosis. A case of pneumococcal endogenous endophthalmitis has been reported in Malaysia that led to a devastating outcome despite both medical and surgical interventions [25].

In general, the treatment modalities for endogenous endophthalmitis were not properly defined in any literature previously and are mainly dependent on the virulence factor [26]. In our case series, our treatment modalities include the commencement of intravenous antibiotics, initiation of early intravitreal tapping, and intravitreal antibiotics upon diagnosis. Subsequently, early pars plana vitrectomy (PPV) initiation depends on the onset, duration of diagnosis, and patient cooperation. Upon establishment of diagnosis, all patients in our center received a minimum of one to three doses of intravitreal injections, combination of intravitreal vancomycin 1mg/0.1ml and intravitreal ceftazidime 2.2mg/0.1ml repeated every 48-72 hours, and initiation of systemic antibiotics such as intravenous ciprofloxacin 400 mg three times daily. Most of the patients were offered early TPPV. However, only one patient agreed. Her vision slightly deteriorated due to cataract in a silicon oil-filled eye.

The role of surgical intervention in endogenous endophthalmitis remains debatable, even though it is still a treatment of choice in sight-threatening bacterial and fungal endogenous endophthalmitis [27]. Vitrectomy was postulated to immediately remove the microorganism load and reduce the ocular structures' inflammatory burden. A study by Yoon et al. concluded that the final visual outcome and prognosis improve with aggressive therapy by early vitrectomy and antibiotic injection [28]. The latest review published in 2020 strongly suggested a therapeutic role of TPPV in patients diagnosed with fungal endogenous endophthalmitis. However, in our review, only one patient agreed to proceed with the TPPV, and due to the limitations of patients diagnosed with fungal endogenous endophthalmitis, we are not able to elaborate more on it.

The final visual prognosis for endogenous endophthalmitis is generally poor, depending on ocular extension [16]. The final visual outcome for our patients ranged from 6/6 to NPL. Two patients improved their final visual acuity; another three deteriorated, with one death reported. One vitrectomized eye had a slight deterioration in vision. However, the patient was able to maintain vision of 6/30. Interestingly, two patients had remarkable improvement in final vision with intravitreal injection alone. All the patients were referred and seen within the same day and commencement of intravitreal tap was done in the same setting upon clinical diagnosis of endogenous endophthalmitis. Yonekawa et al. showed a better visual outcome and prognosis in patients who received early intravitreal injections within 24 hours for bacterial endogenous endophthalmitis [29]. Similarly, Kuo et al. supported the idea that early intervention with intravitreal antibiotics reduced the risk of evisceration. However, no absolute improvement was reported with the commencement of TPPV [26]. In our review, the patient who underwent TPPV did not show marked vision improvement as the patient was diagnosed with *Klebsiella* endogenous endophthalmitis, whereby it is known that *Klebsiella* species is associated with poorer prognosis. This is supported by a review conducted by Khashani and Eliott in their review whereby the *Klebsiella* species has a structure such as polysaccharide capsule which acts as a virulence factor with its highly aggressive disseminated infection prior to first ocular symptoms causing late diagnosis leading to poorer visual outcome [23]. Therefore, early TPPV may not show the best outcome in our patient. In contrast, the role of TPPV may still be the best option in other bacterial or fungal infections, however further study extension should be conducted to understand the nature of this disease as well as its management.

Study limitations

This study was conducted in a newly established tertiary center in Kuantan, Pahang. Therefore, the number of patients is limited. Next, this study is a retrospective case series with a small sample size; thus, some needed data was not adequately available for reporting. Therefore, to expand the study, future prospective studies with longer duration would be more valuable. This study was reported to understand more about the current treatment of endogenous endophthalmitis, whereby lack of proper guidelines in managing endogenous endophthalmitis leads to limitations in this study.

## Conclusions

In this case series of six patients, we observed a variety of outcomes with similar presentations despite standardized treatment in all patients. Five out of six patients showed poorer visual outcomea with only one patient showing final visual acuity of 6/6. Therefore, further study with a larger sample size is needed to evaluate the factors associated with the final visual outcome in endogenous endophthalmitis.
